# Heterogeneity and Potency of Peripheral Glial Cells in Embryonic Development and Adults

**DOI:** 10.3389/fnmol.2022.737949

**Published:** 2022-03-25

**Authors:** Artem Sinegubov, Daria Andreeva, Nikita Burzak, Maria Vasyutina, Lada Murashova, Vyacheslav Dyachuk

**Affiliations:** World-Class Research Centre for Personalized Medicine, Almazov National Medical Research Centre, Saint Petersburg, Russia

**Keywords:** heterogeneity, pluripotency, cellular hierarchy, Schwann cells, neural crest, peripheral glia

## Abstract

This review describes the heterogeneity of peripheral glial cell populations, from the emergence of Schwann cells (SCs) in early development, to their involvement, and that of their derivatives in adult glial populations. We focus on the origin of the first glial precursors from neural crest cells (NCCs), and their ability to differentiate into several cell types during development. We also discuss the heterogeneity of embryonic glia in light of the latest data from genetic tracing and transcriptome analysis. Special attention has been paid to the biology of glial populations in adult animals, by highlighting common features of different glial cell types and molecular differences that modulate their functions. Finally, we consider the communication of glial cells with axons of neurons in normal and pathological conditions. In conclusion, the present review details how information available on glial cell types and their functions in normal and pathological conditions may be utilized in the development of novel therapeutic strategies for the treatment of patients with neurodiseases.

## Embryonic Schwann Cells: Origin, Development, and Stemness

**FIGURE 1:**
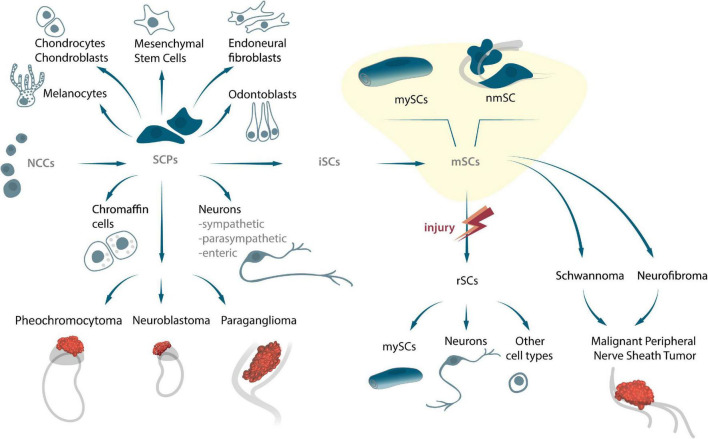
The origin of SCPs and SCs, their potency to differentiate into several cell types, and their capability to transform into peripheral nerve tumors during embryonic and adult stages.

Schwann cells (SCs), or neurolemmocytes, are a type of glial cells that originate from neural crest cells (NCCs). Post delamination from the dorsal neural tube, these cells migrate to the embryo’s body and give rise to multiple cell lineages during early vertebrate development (Le Douarin and Kalcheim, 1999; Bronner and LeDouarin, 2012). NCCs give rise to a wide range of types of differentiated cells, such as craniofacial cartilage and bones, cardiac outflow septum, mesenchyme, pigment cells, and peripheral nervous system (Le Douarin and Kalcheim, 1999). According to the classical view, NCCs specialize into terminally differentiated glia (mature SCs) via a series of intermediate stages. These include, the differentiation of NCCs into Schwann cell precursors (S), followed by the differentiation of SCPs into immature SCs (iSCs), which, in turn, specialize into myelinating (mySCs) or non-myelinating mature SCs (nmSCs) (see review Jessen and Mirsky, 2019). The advent of genetic tracing methods, that allow accurate determination of the hierarchy of crest cells during development, has revealed the complexities associated with the various stages of NCCs differentiation, and that of their descendants. Using Cre-Lox genetic constructions and model animals, have revealed significant insights into the nature of SCPs and their capability to differentiate into a wide range of terminally differentiated cells in early development over the past 10 years (Figure 1; Adameyko et al., 2009; Dyachuk et al., 2014; Kaukua et al., 2014; Furlan et al., 2017; Hockman et al., 2018; Kastriti et al., 2019; Xie et al., 2019; Kamenev et al., 2021). The distinguishing feature that sets SCPs apart from iSCP and mSCPs, is that they retain the ability to differentiate into other cell types (Adameyko et al., 2009; Dyachuk et al., 2014; Kaukua et al., 2014; Furlan et al., 2017; Kastriti et al., 2019; Xie et al., 2019; Kamenev et al., 2021). This characteristic that SCPs partially share with NCCs, may possibly allow them to be exploited for the development of novel therapeutic strategies. Confirmatory evidence of SCPs’ ability to specialize into other cell types was obtained by the demonstration of their differentiation into skin melanocytes (Adameyko et al., 2009) as well as extracutaneous melanocytes to the heart, inner ear, supraorbital locations and brain meninges (Kaucka et al., 2021). Another example of the multipotency of SCPs is their ability to give rise to parasympathetic neurons. Genetic tracing experiments by two independent research groups on transgenic mice, have convincingly demonstrated that parasympathetic neurons in the cranial ganglia, intramular (interstitial) ganglia of the heart, and sacral parasympathetic ganglia after E12.5 are derived from nerve-associated SCPs (Dyachuk et al., 2014; Espinosa-Medina et al., 2014). Genetic tracing has convincingly shown the involvement of local SCPs in origin of the glomus cells of the carotid body oxygen-sensing organ that are primary oxygen-sensing cells (Hockman et al., 2018). Interesting data have been found on the differentiation of SCPs into enteric neurons of the enteric nervous system (ENS). SCPs are capable of differentiating into neurons in the gut during postnatal neurogenesis (Uesaka et al., 2015). Zebrafish lineage tracing performed using lipophilic dyes or the inducible Sox10-Cre system, recently revealed that post-embryonic enteric neurons arise from trunk neural crest-derived SCPs that migrate from the spinal cord into the intestines of these organisms (El-Nachef and Bronner, 2020). Additionally, SCPs might function as a source of mesenchymal cells that produce pulp cells and odontoblasts, as has been observed in a mouse growing tooth model (Kaukua et al., 2014). Besides the above-mentioned cell types, neuroendocrine cells of the adrenal medulla (chromaffin cells) have also been shown to be derived from SCPs in mouse embryos and zebrafish larvae (Furlan et al., 2017; Kamenev et al., 2021). Moreover, chromaffin cells of the organ of Zuckerkandl, and a portion of sympathetic neurons of the posterior paraganglia are known to be largely of SCP origin (Kastriti et al., 2019). Recent genetic lineage tracing has revealed that certain SCPs detach from nerve fibers to become mesenchymal cells, which further differentiate into chondrocytes and mature osteocytes during murine embryonic development. Moreover, the development of chondrocytes from SCPs is also known to occur in zebrafish, thus indicating evolutionary conservation (Xie et al., 2019).

One of the reasons that precluded elucidation of the properties of SCs was the similar expression patterns of key transcriptional factors (TF) including *Sox9/10, FoxD3*, and *Tfap2a/b*, as well as membrane molecules such as *Plp1* and *Erbb2/3* in both SCPs and NCCs (Stewart et al., 2001; Cheung and Briscoe, 2003; Nitzan et al., 2013; Balakrishnan et al., 2016). Despite the molecular similarities between these two cell types, newer data has revealed significant distinguishing characteristics, that are specific to SCPs in the context of cellular and molecular signatures (Lignell et al., 2017; Soldatov et al., 2019).

Several tumors originate from SC lines, which include schwannomas, neurofibromas and malignant tumors of the peripheral nerve membranes (MPNST) (Giovannini et al., 1999; Zhu et al., 2002; Evans et al., 2012; Figure 1). Given the wide potential of SCPs, and the fact that they are direct precursors of these cells under normal circumstances, it is very likely that they are involved in the development of embryonal tumors. For instance, NCCs and SCPs are both the original source of the sympatho-adrenal (sympathetic neurons and chromaffin cells) nervous systems, and can consequently also be responsible for the occurrence of pheochromocytes, neuroblastoma, and paragangliomas during sympatho-adrenal differentiation (Figure 1). The diversity of progenitors and cell transitions during early development might be recapitulated in some solid tumors associated with sympathetic ganglia and the adrenal gland (Lee et al., 2005; Scriba et al., 2020). Recently, using a combination of single cell transcriptomics and lineage tracing it was shown that human intra-adrenal sympathoblasts are directly derived from SCPs and can transit into local neuroendocrine chromaffin cells. Authors suggest, that in humans, this process persists during several weeks of development within the large intra-adrenal ganglia-like structures, which may also serve as reservoirs of originating cells in neuroblastoma (Kameneva et al., 2021). Obviously, new lineage connections might have important implications for understanding of neuroblastoma origin and cell heterogeneity Since multiple tumor cells share key genetic and signaling mechanisms associated with normal development and cancer, an in-depth analysis of the cellular and molecular mechanisms of embryonic cell-cancer cell transitions is of exceeding importance in understanding biological processes and developing personalized medicine.

**FIGURE 2:**
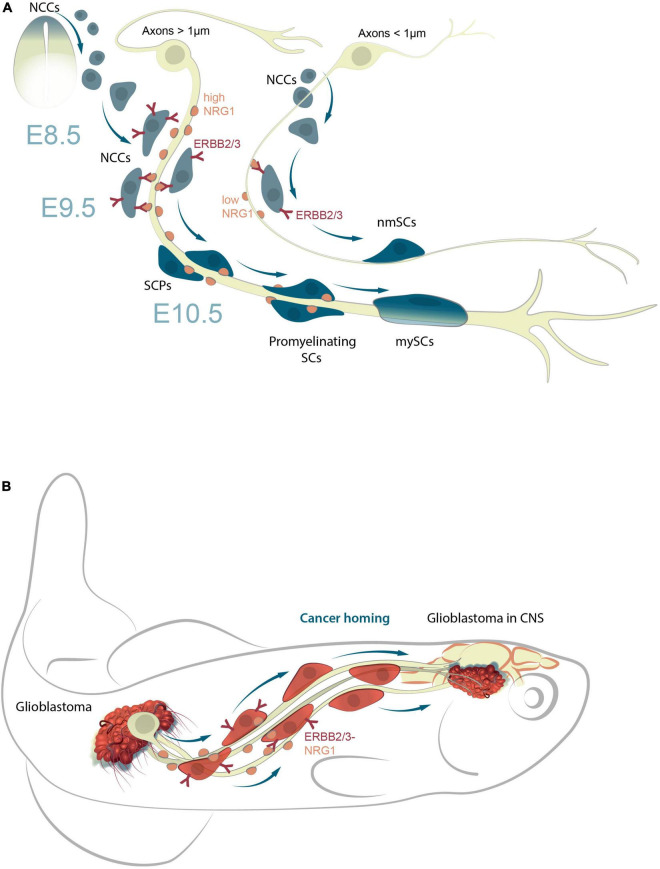
NRG1-ERBB2/3 signal pathway responsible for the maturation of SCPs into mnSCs during development and in cancer homing. **(A)** NRG1-ERBB2/3 interactions are essential for cellular attachment to axons and migration of SCPs along peripheral nerves; SCP survival is dependent on NRG1 concentrations. Those that receive insufficient NRG1 signals undergo apoptosis, or remain NCCs. Those that receive sufficient NRG1 signals differentiate into iSCs and then mySC, or detach from axons on reaching target-tissues and differentiate into other cell types (neurons, chromaffin cells, odontoblasts, or pigment cells). **(B)** A model of NRG1-ERBB2/3 signaling in cancer homing from periphery to CNS (zebrafish model system). NRG1 and ERBB induce the epithelial-mesenchymal transition (EMT) in certain cancers (e.g., breast) by modulating expression of proteins involved in invasion and metastasis. Eventually, the cancer-axonal molecular machinery triggers cancer cell dissemination and homing (e.g., glioblastoma migration from periphery to CNS) via nerve signals.

The mechanisms of attachment NCCs to axons, and their subsequent transformation into SCPs, that either remain attached or detach from nerves to undergo further differentiation into diverse cell types is not fully understood. However, multiple studies on the specialization of glia into different cell types, report a downregulation of the glial program, concomitant with an upregulation of the said cell type program (for instance, neuronal, pigment, or mesodermal programs) (Adameyko et al., 2009; Dyachuk et al., 2014; Xie et al., 2019; Kamenev et al., 2021). The NRG1-ERBB2/3 signaling pathway plays a key role in NCCs and SCP migration/survival in mice and zebrafish (Birchmeier and Nave, 2008; Honjo et al., 2008; Kamenev et al., 2021). The transition from NCCs to SCPs is consequent to the interaction of the former with axons (Figure 2A). The molecular mechanisms that direct a part of the population of NCCs to settle on peripheral nerves, and the underlying reasons responsible for the selection of certain types of nerves are not entirely clear. The transition from SCPs to iSCs is accompanied by an important mechanistic step that involves the selection of axons for myelination (radial sorting) by iSCs, which in turn differentiate into a pro-myelinating SCs (Feltri et al., 2016). The remaining iSCs connect with axons of smaller diameter, and differentiate into non-myelinating SCs (Remak cells) (Monk et al., 2015; Gomez-Sanchez et al., 2017).

The decision of iSCs to differentiate into either myelinating or non-myelinating SCs is dictated by the expression levels of neuregulin 1 (NRG1). While lower levels of NRG1, released by a comparatively smaller axon leads to the maturation of iSCs into non-myelinating SCs, higher levels, result in the development of myelinating SCs (Figure 2A; Michailov et al., 2004; Taveggia et al., 2005; Gomez-Sanchez et al., 2017). Notably, this NRG1-ERBB2/3 signal pathway in human glioma cells, also promotes their migration in malignancy (Zhao and Schachner, 2013). It is likely that this mechanism, is also involved in the migration of cancer cells, for example, when human glioblastomas cells where transplanted into zebrafish (Pudelko et al., 2018; Figure 2B).

Despite the discovery of the ability of SCPs to transform into various cell types, the underlying molecular mechanisms responsible for these transitions remain unelucidated. While the overall pattern of molecular changes evidently involves the downregulation of glial genes and upregulation of cell-specific genes during early development, detailed information on the accompanying changes in gene cascades are not well understood. However, single cell trancriptomic analysis, has led to the revelation of changes in expression patterns of TFs and other molecules, that occur during the transition of NCCS and/or SCPs to specialized cells of the autonomic nervous system, sensory neurons, mesenchymal cells, and chromaffin cells (Dyachuk et al., 2014; Espinosa-Medina et al., 2014; Furlan et al., 2017; Soldatov et al., 2019). This data on the genetic basis of cellular transitions in early development might contribute immensely to the development of novel technologies, which may be exploited to regulate the processes of differentiation during development, or to correct human pathological states. Currently, several issues pertaining to the heterogeneity of SCPs and iSCs during developmental processes need to be addressed. These include unraveling the extent of heterogeneity among SCPs located on different nerves. Further, questions regarding how the positions of nerves, and glia attached to nerves, might affect the molecular signatures of SCPs need to be answered.

A secondary embryonic glial cell population, referred to as boundary cap cells (BCCs) is a subpopulation of multipotent cells that originate from NCCs, and give rise to cells of different lineages (Golding and Cohen, 1997). BCCs contribute with all SCPs occupying the dorsal roots, the progenitors of neurons, mainly nociceptive afferents, and satellite cells (Maro et al., 2004). Besides, BCC migrate along peripheral nerves to reach the skin, where they give rise to terminal glia associated with dermal nerve endings and neurogenic stem cells in the skin (Gresset et al., 2015). BCCs are localized to the dorsal root entry zone and motor exit point of the embryonic spinal cord, at the border between the central and peripheral nervous systems (Niederländer and Lumsden, 1996; Radomska and Topilko, 2017). Microarrays, genetic deletion assays, and single cell transcriptomic analysis have revealed sets of TFs that are expressed in both NCCs and BCCs (*FOXD3* and *SOX10*), along with BCCs -specific TFs (*KROX-20, NTRN5, WIF1*, and *HEY2*) (Coulpier et al., 2009; Garrett et al., 2016; Faure et al., 2020). Supportive evidence for the multipotency of BCCs has been provided by genetic tracing and mice knockout models. These studies have determined that the functions of these cells include the production of glia and neurons of the PNS, as well as skin melanocytes and pericytes in cutaneous vasculature. Additionally, they also serve as gatekeepers that prevent motor neurons from escaping the CNS (Radomska and Topilko, 2017).

## Adult Peripheral Glial Cells: Heterogeneity, Proliferation, Stemness, and Repair Capacity

**FIGURE 3:**
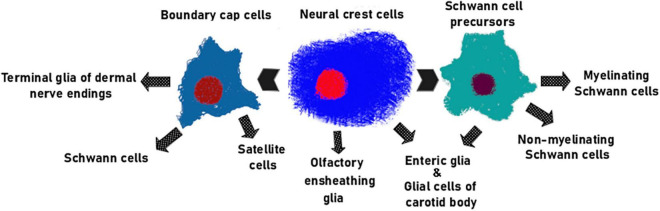
The main glial lineages originating from NC. Olfactory ensheathing cells (Barraud et al., 2010; Murdoch et al., 2010; Forni et al., 2011; Katoh et al., 2011) and majority of enteric glia (Espinosa-Medina et al., 2017) are direct descendants of NCCs. SCPs generate both myelinating and non-myelinating SCs (Jessen and Mirsky, 2019) along with portion of enteric glial cells (Uesaka et al., 2021). BCCs give rise to satellite glia (Maro et al., 2004), glomus cells of the carotid body (Hockman et al., 2018), terminal glial cells of the dermal nerve endings (Gresset et al., 2015) and mature Schwann cells of the nerve roots (Coulpier et al., 2009).

Neural crest cells generate a diverse set of specialized non-myelinating cells during prenatal development. These include satellite glial cells of sensory and autonomic ganglia (Zirlinger et al., 2002; Dyachuk et al., 2014; Furlan et al., 2017), olfactory ensheathing cells (OSNs) (Barraud et al., 2010), perisynaptic Schwann cells (PSCs) (Armati, 2007), enteric glia (see review Bronner and LeDouarin, 2012), and other uncharacterized subtypes of adult glial cells (Figure 3). The following section of this review focuses on the biology and function of those populations of adult glia cells that may be potentially exploited for the regeneration of axons and other different cell types, and thereby aid the development of cell-based therapies to treat various pathologies.

### Perisynaptic Schwann Cells

Perisynaptic Schwann cells (PSCs) are localized at neuromuscular junctions and play a key role in synapse formation during development (Herrera et al., 2000). Although growing motor axons successfully reach target muscles and inhibition of muscle activity allows synapse formation even in the complete absence of PSCs (Lin et al., 2000; Liu et al., 2019), they are essential to prevent the degeneration of nerve terminals after initial contact (Riethmacher et al., 1997). PSCs participate in the remodeling of motor units during early postnatal development. The process involves the initial formation of multiple connections between each muscle fiber with several motor neurons, and the subsequent transition to mononeural innervation by the separation and elimination of competing nerve terminals is mediated by PSCs. The decoding of presynaptic activity by PSCs has been attributed to purinergic signaling (Darabid et al., 2013). Thus, both the synaptic efficacy of competing terminals and postsynaptic activity play key roles in the regulation of synapse elimination by PSCs.

The function of the PSC is not limited only to clearing neuronal debris during early development. PSCs have been demonstrated to function as major phagocytic cells such disease as Guillain-Barre syndrome (Cunningham et al., 2020). After denervation, PSCs formed “bridge-like” structures with undamaged synapses (Love and Thompson, 1999), which facilitate the growth of nerve terminals to denervated endplates. The sprouting of PSC is dependent on NRG1-ErbB2 signaling, since both the expression of the constitutively active ErbB2 receptors (Hayworth et al., 2006), and the presence of the soluble form of neurogenin (GGF2), enhance the migratory potential of these cells (Trachtenberg and Thompson, 1997). Further, the treatment of chicken embryo with the NRG antagonist HBD-S-H4, has been shown to significantly attenuate the encapsulation of synapses by PSCs (Wang et al., 2017).

In the early stages of synaptogenesis, PSCs connect with pre-patterned acetylcholine receptor clusters that are localized near nerve endings (Barik et al., 2016). Several factors, including TGF-β1 (Feng and Ko, 2008a), BDNF (Feng and Ko, 2008b), as well as the B11 and B19 isoform of agrins (Yang et al., 2001), that are produced by PSCs support the formation of presynaptic terminals. PSCs are also capable of argins cleavage via the secretion of matrix metalloproteinase 3 (VanSaun et al., 2007). MMP3 KO mice have been observed to develop a larger number of junctional folds, and a higher density of the acetylcholine receptor clusters in comparison to those of the wild-type (VanSaun et al., 2003). These factors also play a role in long-term synapse maintenance. While acute ablation of PSCs in adult mice did not induce neuromuscular dysfunction, it was found to cause a severe reduction in neurotransmitter release, and axon viability on day 6 after loss of glial support (Todd et al., 2010).

Abnormal functioning of PSCs has been observed in SOD1*G*37*^R^* mice, which are a model for slow-onset amyotrophic lateral sclerosis. In normal physiological conditions, PSCs detect synaptic activity due to an increase in intracellular Ca^2+^ concentration. SOD1*G*37*^R^* mice have abnormally enhanced intracellular Ca^2+^ concentrations, even before the onset of denervation (Martineau et al., 2020). The loss of S100-positive cells that is induced by nerve crush injury, is more pronounced in this strain, and is consequently associated with delayed reinnervation and functional recovery (Carrasco et al., 2016). Furthermore, the intrusion of morphologically abnormal PSCs in the synaptic cleft has been identified in muscle biopsies from patients with amyotrophic lateral sclerosis (Bruneteau et al., 2015).

### Olfactory Ensheathing Cells

Postnatal neurogenesis is known to occur in the olfactory neuroepithelium. Given that olfactory sensory neurons (OSNs) have an average lifespan of 1–3 months (Ekberg et al., 2012), extensive proliferation and differentiation of neural stem cells are required to match the turnover rate. A unique type of cells referred to as olfactory ensheathing cells (OECs), that cannot unambiguously be attributed to any major glial type, are known to play a crucial role in axon extension and guidance during this process (Boyd et al., 2005).

The majority of cell types that populate the peripheral part of the olfactory system are descendants of the olfactory placode (Forni et al., 2011). Although OECs migrate along with nascent olfactory fibers as part of a migratory mass, their neural crest origin was determined by genetic lineage tracing by using *Pax7^Cre^* (Murdoch et al., 2010), *Wnt1^Cre^* (Barraud et al., 2010; Forni et al., 2011; Katoh et al., 2011), and *P0^Cre^* (Katoh et al., 2011) mice. Although the expression profile of OECs shares some similarities with that of SCs (Perera et al., 2020), functionally however, these cells encase bundles of multiple axons rather than form myelin sheaths (Murtaza et al., 2019). Another distinctive characteristic of OECs, is their ability to actively migrate from the PNS into the CNS (Reshamwala et al., 2019). During development, as well as in continuous postnatal remodeling, these cells are known to migrate ahead of a growing axon toward the glomerular layer of the olfactory bulb (Ekberg et al., 2012). While direct contact between the growth cone and OEC processes is believed to be critical, their reparative potential may be also be attributed in part to distant intercellular interactions, and the maintenance of a suitable microenvironment. In fact, OECs-conditioned media have been shown to induce axonal growth *in vitro* and *in vivo* (Gu et al., 2016).

Extracellular matrix proteins and various neurotrophic factors are potential mediators of the above-mentioned effect. OECs express a variety of neurotrophic factors, including that referred to as axogenic NGF (Cao et al., 2007), BDNF (Pastrana et al., 2007), CNTF (Wewetzer et al., 2001), NT-4, and GDNF (Cao et al., 2004). Their actions, however, are not limited only to their effect on axons, since these cells simultaneously also express a range of receptors for the same factors. GDNF has been shown to enhance the motility of OECs by the activation of JNK and Src via the GFRα1 and RET receptors (Cao et al., 2006). An experimental GDNF over-secreting cell line was found to be superior to wild-type OECs in promoting axonal extension (Cao et al., 2004). Additionally, FZD1 and NRP1 may be potential scavengers of WNT1 and SEMA3A, that are recognized inhibitors of axonal growth, after spinal cord lesions (Perera et al., 2020). Extracellular vesicles isolated from human OECs are known to increase proliferation of neural progenitors, inhibit oxidative stress-induced neuronal toxicity *in vitro* (Tu and Hsueh, 2020), and promote regeneration of peripheral nerves *in vivo* (Xia et al., 2019).

Olfactory ensheathing cells are have been shown to modulate axonal regeneration via microenvironment modification. These cells function as major phagocytic cells in the olfactory epithelium and olfactory bulb, to remove neuronal debris (Nazareth et al., 2014). Further, they retain this property even at their site of transplantation (He et al., 2014). High-throughput proteomics has identified at least 168 extracellular proteins that are secreted by cultured rat OECs (Liu et al., 2010), which include collagen type IV, fibronectin, and laminin amongst others. OECs also express a range of adhesion proteins, including E-NCAM (Barnett, 2004), L1, β2-laminin, and N-cadherin (Fairless et al., 2005), that are essential for their migration and axonal guidance. Knockdown of genes *LEPRE1* and *NID2* encoding collagen and laminin-binding proteins, resulted in suppression of neurite outgrowth in a co-culture of OECs and DRG neurons (Wassenhove-McCarthy and McCarthy, 1999). Further, these cells reduce the expression of chondroitin sulfate proteoglycans in excessively proliferating glial cells (Wang G. Y. et al., 2021).

Olfactory ensheathing cells are also known to possess immunomodulatory properties. After grafting them into the sub-retinal space, they have been shown to reduce the rate of retinal degeneration via downregulation of the Notch signaling pathway in Müller cells (Xie et al., 2017). Moreover, their anti-inflammatory effect after intravenous transplantation in injured spinal cord has been conclusively demonstrated (Zhang et al., 2021).

In recent decades, the unique properties of OECs that promote axonal growth have driven the development of OEC-based cell therapies for CNS trauma. Pre-clinical studies on OEC transplantation, for the treatment of spinal cord injury has been summarized in several systematic reviews and meta-analyses (Liu et al., 2014; Li et al., 2015; Watzlawick et al., 2016; Nakhjavan-Shahraki et al., 2018). Overall, the process seems to effectively restore motor function, however, the recovery of sensory function has been less successful. The inconsistent outcomes may be accounted for in part, by differential purities of cell preparations and the variabilities introduced by different sources of OECs. Nevertheless, preparations from the olfactory bulb typically have more stable cell composition (Barnett and Chang, 2004), and OECs derived from the mucosa have been found to be most beneficial for this application (Jani and Raisman, 2004), especially since obtaining olfactory mucosal biopsies is a simple and non-invasive technique. Various preparations have been used thus far for transplantation in clinical trials, that range from OECs that have been purified by selective media (Bianco et al., 2004) to whole tissue pieces of undetermined cellular composition (Chhabra et al., 2009).

Olfactory ensheathing cell transplantation has remarkable potential to enhance peripheral nerve regeneration and remyelination after transection. These cells were found to form myelin sheaths on regenerated axons distal to the transection site after microsurgical reparation (Radtke et al., 2008). Further, reparation with OECs embedded in a polycaprolactone conduit was found to be as effective as an autologous nerve graft (Donoghue et al., 2013).

However, the bridging of large nerve defects requires the utilization of different materials for the preparation of artificial nerve grafts that are seeded with OECs. Examples of these include, PLGA (Li et al., 2010), acellularized vein filled with spider silk (Radtke et al., 2011), muscle-stuffed vein (Lokanathan et al., 2014), collagen (Goulart et al., 2016), as well as silicone and nanofibrous CNT/PLLA (Kabiri et al., 2015).

### Satellite Glia

Satellite glial cells (SGCs) are found in ganglia (sensory, sympathetic, and parasympathetic ganglia) of the peripheral nervous system (PNS) (see review Hanani, 2010). These cells surround neuronal cell bodies. SGCs express several markers including cadherin 19 (George et al., 2018), potassium channel Kir4.1 (Vit et al., 2008), glutamine synthetase (GS) (Miller et al., 2002), and GFAP (Wang et al., 2019). Each neuron is usually isolated from those surrounding it by several enveloping glial cells that create a suitable microenvironment (Cece et al., 1995). Neuroglial communication in sensory ganglia occurs predominantly via chemical messengers. SCs express a plethora of functional receptors that are involved in the regulation of pain sensitivity, including purinergic receptors (Villa et al., 2010), the NMDA receptor (Castillo et al., 2013), TRPA1 (Shin et al., 2020), and CGRP receptors (Edvinsson et al., 2020). However, communication between cells in ganglia is also mediated via direct contact. In normal physiological conditions, satellite cells express Cx26, Cx47 (Garrett and Durham, 2008), and Panx1 (Retamal et al., 2014; Zhang et al., 2015). These proteins form gap junctions and membrane channels that allow direct diffusion of small molecules between the intracellular compartments of neighboring cells. It has been previously suggested, that the formation of interconnected cell clusters may play a crucial role in hyperalgesia and its associated pathologies. This notion is supported by the observation of abnormally increased coupling of SCs near gap junctions in various models of chronic pain and inflammation (Zhang et al., 2015; Blum et al., 2017; Komiya et al., 2018; Yamakita et al., 2018). The inhibition of SC-SC and SC-neuron coupling has been demonstrated to be a potential target for pain management. Knockdown of the most abundant connexin in mouse SCs, *Cx43*, has been shown to reduce pain post nerve injury (Ohara et al., 2008). The gap-junction blocker carbenoxolone has also demonstrated analgesic properties in acute pain models (Lemes et al., 2018). Direct electrical coupling between SCs and neurons has been observed in cultured (Suadicani et al., 2010) and freshly dissected sensory ganglia (Spray et al., 2019), and the association between coupled activation of DRG neurons and the upregulation of gap junctions has been shown in a mouse model of inflammation and nerve injury (Kim et al., 2016). Neuronal activity leads to the release of cytokines (TNF, IL-1β, and IL-6) by SCs, which increases neuronal excitability (Dubový et al., 2006). An instance of such altered secretion was observed as a consequence of pancreatic cell destruction, which contributed to the pathogenesis of diabetic neuropathic pain (Gonçalves et al., 2018).

While little is known about the biology of SGCs, it is becoming increasingly obvious that these cells on account of being localized in morphologically different neuro-structures, possess distinct molecular, morphological, and functional signatures. Moreover, recent data from a single cell RNA sequencing (scRNAseq) study has permitted the analysis of the molecular profiles of these cells in various physiological processes, including the development of somatosensory dorsal root ganglia and auditory spiral ganglia (Tasdemir-Yilmaz et al., 2021), in individual types of adult ganglia (Avraham et al., 2020; van Weperen et al., 2021), in nerve regeneration (Avraham et al., 2020), and pain (Wang K. et al., 2021). scRNAseq data analysis of several types of ganglia convincingly demonstrated a high level of SGCs heterogeneity in sympathetic stellate ganglia (van Weperen et al., 2021) and also in both naïve and injured conditions (Avraham et al., 2020). The transcriptomic profiles of SGCs in the stellate ganglia revealed the presence of five subpopulations (by degree of maturity) of SGCs, that shared many characteristics, including common signaling and metabolic pathways (van Weperen et al., 2021). scRNAseq data of adult L4-L5 DRG (in control mice) identified 13 distinct cell clusters among which SGCs, with high expression of *Kir4.1*, *Cdh19*, and *Bfabp*, but not the classical marker *Glul/GS*, accounted for 28% of the total cells. In the same analysis, SCs with high expression of *Mpz, Mbp, Plp1, Mag, Prx*, and *Ncmap* accounted for 10% of the adult DRG (Avraham et al., 2020). Despite the fact that the scRNAseq data obtained from different ganglia of the PNS, is insufficient for in-depth comparison and identification of the extent of glia heterogeneity, data from sympathetic ganglia and DRG have revealed that glia are highly heterogeneous, and are characterized by both unique and common molecular markers.

### Enteric Glia

Enteric glial cells (EGCs) comprise a large population of peripheral glia of the enteric nervous system (ENS), that are part of ganglionated myenteric and submucosal plexus (myenteric/Auerbach and submucosal/Meissner) and extra ganglionic spaces, within the intestinal mucosa and muscle layers (Gershon and Rothman, 1991). These cells have been classified into four subgroups based on differences in cellular morphology (Hanani, 1994). They have also been classified based on their locations in the intestinal wall (mucosal, intraamniotic, and intramuscular intestinal glia) (Gulbransen and Sharkey, 2012). EGCs express proteins that are characteristic of other types of glia. For instance, they express SOX10, GFAP, PLP1, and S100beta I​ /​ I as has been observed in astrocytes (Jessen and Mirsky, 1983; Boesmans et al., 2015). Their TFs expression profile however, is similar to that of myelinating glia, and not of astrocytes (Rao et al., 2015). SOX10 and PLP1 are expressed in myelinating glia (Schwann cells) and oligodendrocytes, and in conjunction with S100B are common markers that are used to characterize EGCs (Hoff et al., 2008; Rao et al., 2015). Given that EGCs and SCs share some TFs, it is difficult to distinguish between them in the ENS (Rao et al., 2015). Specialized functions and specific locations in the tissues/organs of the digestive system add a heterogeneity imprint on EGCs, leading to the appearance of individual subpopulations with unique characteristics. However, little is known about the local heterogeneity of EGCs and the functional aspects managed by these subpopulations in the regulation of digestion and homeostasis.

Recent transcriptional profiling data revealed that EGCs diversity differs between regions of the digestive tract, and that the ENS glial diversity of humans, is greater than that of mice (Zeisel et al., 2018; Drokhlyansky et al., 2020). A scRNA-seq study of the Wnt1Cre;R26RTomato transgenic mouse nervous system identified seven EGCs subtypes in the small intestinal myenteric plexus (Zeisel et al., 2018). The problem of separating SCs from EGCs was tackled by adopting the single-cell transcriptional approach, which revealed that the expression patterns of individual genes, including *Dhh, Mal*, and *Mpz* can distinguish two cell types that express similar TFs (Zeisel et al., 2018; Morarach et al., 2021). Several enteric glial populations with unique transcriptional profiles have been described in the adult mouse and human ENS using newer methods that isolate cell nuclei from the gut for RNA expression analysis (Drokhlyansky et al., 2020). Three transcriptionally distinct populations of colonic EGCs have been identified based on the expression of *Gfra2*, *Slc18a2*, and *Ntsr1* in mice, while the human colon was found to contain six glial subsets (Drokhlyansky et al., 2020). Despite greater understanding of the diversification of glial cell populations of the ENS, the role of transcriptional molecules in the spatial and morphological diversity of EGC groups, remains unknown. Further research on the comparative analysis of EGC with other populations of peripheral glia, including satellite glia, sympathetic and parasympathetic glia composed of ganglia, intramular ganglia, and regional myelinized and non-myelinized SCs will aid comprehension of cellular diversity and function.

### Repair Schwann Cells

Repair Schwann cells (rSCs) are a separate population of adult SCs, that lose contact with and demyelinate the distal stump after axonal injury. These cells undergo reprogramming that involves the upregulation of several genes and activation of multiple transcriptional mechanisms. For instance, rSCs are known to upregulate c-Jun, mitogen-activated protein kinase (MAPK) pathway, Sonic Hedgehog (Shh) pathway, secrete trophic factors that support the survival of damaged neurons, and promote axonal regrowth (Arthur-Farraj et al., 2012; Nocera and Jacob, 2020). This process occurs via a transcriptional program orchestrated by c-JUN that culminates in the generation of “repair” glial cells with a specific molecular signature (Arthur-Farraj et al., 2012). The TFs expression patterns of these cells share commonalities with that of epithelial-mesenchymal transition (EMT) (Arthur-Farraj et al., 2017; Clements et al., 2017; Jessen and Arthur-Farraj, 2019).

## Potency of Adult Peripheral Glial Cells to Differentiate Into Another Cell Types in Normal and Pathological Conditions

Investigations into the ability of embryonic glia to differentiate into different cell types, has led to further studies that explore the possibility of similar properties in adult glia (Falk and Götz, 2017). While multiple data on the ability of differentiated glia to transform into various cell types are available, the range of such transformations is severely limited in comparison to that of embryonic glia. Despite the presence of different subpopulations of glial cells in the PNS, not all are capable of active proliferation and differentiation. For instance, the major cell types of peripheral glia, namely, myelinating SCs (mySCs) and non-myelinating SCs (nmySCs) are inert cell that rarely undergo division in normal circumstances (Nocera and Jacob, 2020). After injury, however, all mySCs are capable of dedifferentiating into proliferative progenitor-like SCs, that subsequently transform into mSCs cells, which orchestrate regenerative responses. These processes occur without any contribution from regional stem cells, and consequently the recovery process is limited by its own capacity and by neuro-glial signals. Dedifferentiated glial cells, such as repair SCs (rSCs) are poorly understood despite the fact that their specific molecular signatures are well defined. Evidence suggests that a significant expansion of Sox2+, S100b+ dedifferentiated SCs within the regenerating digit coincident with blastema growth in the model of Sox2-*CreERT*^2^/+; R26-*^lsl–TdTomato^* mice (Arnold et al., 2011) is necessary for normal nail and bone regeneration (Johnston et al., 2016), and for skin regeneration via the forceful proliferation of mesenchymal cells (Johnston et al., 2013). Peripheral glial cells are known to be activated in response to injury, and promote wound healing in the skin of adult mice. The process is initiated by the detachment of SCs from injured nerves, which then proceed to move into the granulation tissue, where they undergo reprogramming into invasive mesenchymal-like cells, and ultimately drive peripheral nerve regeneration (Clements et al., 2017; Parfejevs et al., 2018). All of the above-mentioned examples describe the indirect effects of glia on other cell types and tissues in response to damage.

Recent data on zebrafish reveals that enteric neurons do not originate from resident neuronal progenitors or enteric glia that are absent in adult zebrafish, but from trunk neural crest-derived SCPs that migrate into the intestine, and differentiate into neurons. This indicates a new role for SCPs in the context of adult neurogenesis, where they function as a source of enteric neurons in adult fish and mouse (Uesaka et al., 2015; Espinosa-Medina et al., 2017; El-Nachef and Bronner, 2020; Kamenev et al., 2021). These are the only examples of post-embryonic SCPs that differentiate into distinct types of neuronal or non-neuronal cells that have been recorded till date.

## Future Perspectives

Despite documentation of the distinguishing characteristics of SCPs, the mechanisms that operate to select their fate, and direct them to specialize into certain cell types (neurons, neuroendocrine cells, odontoblasts, pigment cells, osteoblasts, and osteoclasts) are poorly understood. Some of the questions that need to be answered include: (1) What causes a cell to detach from a nerve and undergo differentiation? (2) Do the transforming signals arrive from peripheral nerves (positional information) on which glia are located? (3) Could they possibly arise from the internal heterogeneity of SCPs, from neighboring tissues, or all of the above? A second aspect that need to be studied is the application of the multipotency of SCPs in clinical scenarios. Disturbances in the migration and directed differentiation mechanisms of SCPs can result in a wide range of disorders associated with lack of sufficient cells required for proper functioning. This further leads to abnormalities in the development of tissues/organs, including the parasympathetic ganglia and their innervations, neurogenesis of the enteric system, adrenal glands, skeletal structures, teeth, and pigmentation. For instance, more than 20 types of autonomic dysfunctions currently known, cause symptoms including heartburn, intestinal gas, flatulence, diarrhea, constipation, colitis, dry mouth, cardiac disorders, dysuria, and sexual dysfunction. The mechanisms that underlie these disorders have not been elucidated, but may possibly involve SCPs and their ability to differentiate into autonomic neurons. The immense potencies of different glia types in adults are just beginning to be discovered. Cells of several peripheral glia types have been found to be capable of differentiating into neurons under both normal and pathological conditions, participating in perineural invasion, and playing important roles in carcinogenesis, apart from their involvement in axon regeneration.

## Author Contributions

AS, DA, NB, MV, LM, and VD planned the study, and wrote the manuscript. MV and LM participated in data acquisition. VD supervised the study. All authors read and approved the final version of the manuscript.

## Conflict of Interest

The authors declare that the research was conducted in the absence of any commercial or financial relationships that could be construed as a potential conflict of interest.

## Publisher’s Note

All claims expressed in this article are solely those of the authors and do not necessarily represent those of their affiliated organizations, or those of the publisher, the editors and the reviewers. Any product that may be evaluated in this article, or claim that may be made by its manufacturer, is not guaranteed or endorsed by the publisher.
